# Investigation and management of hypocalcaemia amongst critically ill patients

**DOI:** 10.1186/cc9790

**Published:** 2011-03-11

**Authors:** A Carins, M Mogk, ID Welters

**Affiliations:** 1 Royal Liverpool University Hospital, Liverpool, UK; 2 Moredata GmbH, Giessen, Germany

## Introduction

There is a growing body of evidence linking the presence of hypocalcaemia with greater morbidity and mortality in the critically ill [1]. At present, no national guidelines for the treatment of hypocalcaemia in critically ill patients exist. The purpose of this investigation was to determine the prevalence of hypocalcaemia on admission to critical care, to assess the current diagnosis and treatment regime and to attempt to identify any correlation between severity of illness and the prevalence of hypocalcaemia.

## Methods

Data were collected for all patients admitted to a 13-bed ICU of a tertiary referral centre in September 2010 for at least three consecutive days of their stay. Three patients were subsequently excluded, as their data were incomplete. Serum and ionized calcium levels were reviewed for the presence of hypocalcaemia on admission and evidence of improvement over time. Sepsis was assessed according to the ACCP/SCCM Consensus definitions and APACHE II scores were calculated. Calcium levels were compared using the Wilcoxon test.

## Results

Fifty-three patients, 62% men and 38% women, were included. Ionized calcium levels on admission showed 75.0% of patients to be hypocalcaemic, while serum calcium levels revealed hypocalcaemia in only 72.6%. Supplementation of calcium gluconate based on daily serum calcium levels was found to be an effective treatment for hypocalcaemia and led to a significant increase in both ionized and serum calcium concentrations on day 3 (*P *= 0.001 and 0.020). On the third day of their stay on the ICU, 43.1% and 34.7% of patients still had low ionized and serum calcium levels. Serum calcium levels generally mirrored ionized calcium levels; however, compared with ionized calcium levels, hypocalcaemia remained undetected in two out of 53 patients (3.8%). There was no correlation between the severity of disease and the occurrence of hypocalcaemia. Similarly, a diagnosis of sepsis, severe sepsis and septic shock was not associated with hypocalcaemia.

## Conclusions

Serum calcium levels tend to underestimate hypocalcaemia compared with ionized calcium. Although the existing treatment strategy was found to be effective in general, the use of ionized calcium levels for detection and treatment of hypocalcaemia might be more effective [2].
